# *N*-(5-Mercapto-1,3,4-Thiadiazol-2-yl)-2-Phenylacetamide Derivatives: Synthesis and *In-vitro* Cytotoxicity Evaluation as Potential Anticancer Agents

**Published:** 2014

**Authors:** Ahmad Mohammadi-Farani, Neda Heidarian, Alireza Aliabadi

**Affiliations:** a *Department of Pharmacology, Toxicology and Medical Services, Faculty of Pharmacy, Kermanshah University of Medical Sciences, Kermanshah, Iran. *; b *Students Research Committee, Kermanshah University of Medical Sciences, Kermanshah, Iran. *; c *Department of Medicinal Chemistry, Faculty of Pharmacy, Kermanshah University of Medical Sciences, Kermanshah, Iran.*

**Keywords:** Synthesis, 1, 3, 4-Thiadiazole, Anticancer, Amidation

## Abstract

A new series of *N*-(5-Mercapto-1,3,4-thiadiazol-2-yl)-2-phenylacetamide derivatives (3a-3j) were synthesized via an amidation reaction using EDC and HOBt in acetonitrile solvent at room temperature condition. Chemical structures were characterized by ^1^H NMR, IR and MS spectroscopic methods and related melting points were also determined. The anticancer activity was evaluated using MTT procedure *in-vitro*. All compounds were tested against SKNMC (Neuroblastoma), HT-29 (Colon cancer) and PC3 (Prostate cancer) cell lines. According to the toxicological data, none of the synthesized derivatives exerted superior activity than doxorubicin as reference drug. Derivatives with *Ortho* chlorine (compound 3d), *meta* methoxy (compound 3h) and *meta* fluorine (compound 3b) substituents on the phenyl ring exhibited the best cytotoxic activity against SKNMC (IC_50_ = 4.5 ± 0.035 µM), HT-29 (IC_50_ = 3.1 ± 0.030 µM) and PC3 (IC_50_ = 12.6 ± 0.302 µM) cell lines respectively.

## Introduction

Cancer is a major problem throughout the world and is the second leading cause of mortality in developed countries. Since, many of the current treatments have problems with adverse effects and drug-resistance; there is a strong and vital demand for the discovery and development of effective new anticancer therapeutics. Currently, cancer is the second leading cause of death in the developed countries. Tremendous progress has been made in the war against cancer with the development of many novel chemotherapy agents. The main treatments for neoplastic diseases involve surgery, chemotherapy and radiotherapy. Chemotherapy involves the use of low-molecular-weight drugs (doxorubicin, methotrexate, paclitaxel,…) to selectively destroy tumor cells or at least limit their proliferation. Disadvantages of many anticancer agents include myelosuppression, gastrointestinal side effects (nausea and vomiting), hair loss (alopecia), and also the development of clinical resistance (1-7).

**Figure 1:**
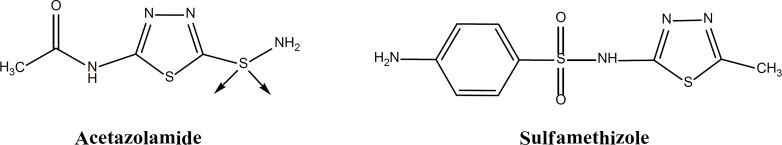
Structures of acetazolamide (carbonic anhydrase inhibitor) and sulfamethizole (antibacterial agent) as examples of drugs containing 1,3,4-thidiazole ring

During recent years a wide and intense investigation of different pharmacophores and chemical classes containing 1,3,4-thiadiazole have been carried out. Many of these derivatives possess interesting and potential biological effects such as antimicrobial, antitubercular, antiviral, anti-inflammatory, anticonvulsant, antihypertensive, antioxidant, antifungal and anticancer activity and now there are in the market as common used drugs (Figure 1) (8-16).

**Figure 2:**
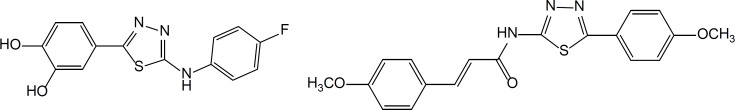
Structures of two 1,3,4-thiadiazole based compounds with potential anticancer activity.

Diverse chemical structures containing 1,3,4-Thiadiazole nucleus have been reported with potential anticancer activity (Figure 2). The 1,3,4-thiadiazole ring in anticancer agents performs its role in pharmacophroes of apoptosis inducers and caspase activators, tyrosine kinase inhibitors, carbonic anhydrase inhibitors and *etc* (17-25). Hence, various mechanisms could be imagined for anticancer chemical structures that containing the 1,3,4-thiadiazole ring.

In the present study, we focused on the synthesis of novel thiol containing 1,3,4-thiadiazole derivatives and assessed their anticancer activity against three cancerous cell lines consist of PC3 (Prostate cancer), HT-29 (Colon cancer) and SKNMC (Neuroblastoma). 

## Results and Discussion

*Chemistry*

**Scheme 1:**
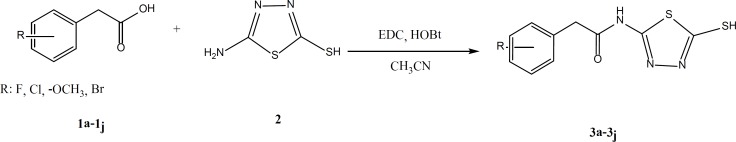
Synthetic procedure of compounds 3a-3j.

According to the scheme 1, 5-amino-1,3,4-thiadiazole-2-thiol (1) was treated directly with various derivatives of phenylacetic acid for amide bond formation. The reaction was carried out in the presence of EDC and hydroxybenzotriazole (HOBt) in acetonitrile as solvent. The completion of reaction was confirmed by thin layer chromatography (TLC). After completion, the solvent was evaporated under reduced pressure and ethyl acetate and water were added. The aqueous phase was separated and the organic phase was washed two times by sodium bicarbonate 5%, diluted sulfuric acid and brine (26, 27). Anhydrous sodium sulfate was added and filtration was done. Ethyl acetate was removed using rotatory evaporator and the intended product was obtained as powder.

*MTT assay*

According to the Table 1, cytotoxicity of all synthesized compounds 3a-3j was evaluated against three cancerous cell lines using MTT method. PC3 (Prostate cancer), HT-29 (Colon cancer) and SKNMC (Neuroblastoma) was applied in this investigation. None of the synthesized compounds showed superior activity than doxorubicin as reference drug. Totally, all compounds exhibited a higher activity against PC3 and HT-29 cell lines in comparison with SKNMC. Fluorine substituent at position *meta* (compound 3b) demonstrated the best anticancer activity against SKNMC cell line. This pattern was also observed for PC3 cell line. But, *para* substitution (compound 3c) of fluorine afforded a better activity against HT-29 cell line. Chlorine moiety at position 2 of the phenyl ring rendered a high anticancer potency against SKNMC cell line (IC_50_ = 4.5 ± 0.035 µM). Substitution of the methoxy group on the phenyl ring enhanced the activity against HT-29 cell line especially at positions *ortho* and *meta* (compound 3g and 3h). *Ortho* position of the methoxy moiety was also favorable for cytotoxic activity against SKNMC and PC3 cell lines (compound 3 g). Substitution of bromine atom at position 4 of the phenyl ring caused a better anticancer activity against SKNMC cell line compared to HT-29 and PC3 cell line. 

**Table 1:** Cytotoxicity results of compounds 3a-3j against three cancerous cell lines (SKNMC: neuroblastoma, HT-29: colon cancer, PC3: prostate cancer). Results were represented as IC_50_ (µM) values.

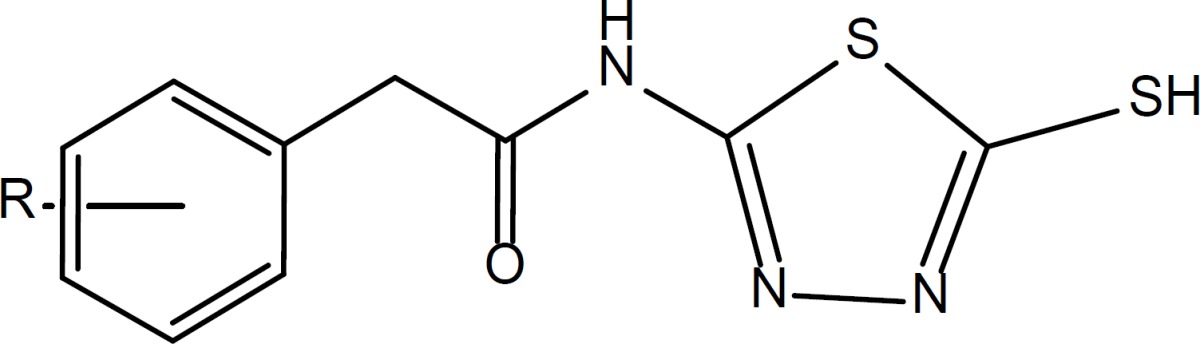

## Experimental

*Chemistry*

All starting materials, reagents and solvents were purchased from commercial suppliers like Merck and Aldrich companies. The purity of the prepared compounds was proved by thin layer chromatography (TLC) using various solvents of different polarities. Merck silica gel 60 F_254_ plates were applied for analytical TLC. Column chromatography was performed on Merck silica gel (70-230 mesh) for purification of intermediate and final compounds. ^1^H-NMR spectra were recorded using a Varian 400 MHz and Bruker 200 MHz spectrometer, and chemical shifts are expressed as δ (ppm) with tetramethylsilane (TMS) as internal standard. The IR spectra were obtained on a Shimadzu 470 spectrophotometer (potassium bromide disks). Melting points were determined using electrothermal melting point analyzer apparatus and are uncorrected. The mass spectra were run on a Finigan TSQ-70 spectrometer (Finigan, USA) at 70 eV. All cell lines were purchased from Pasteur Institute of Iran.

*General procedure for synthesis of N-(5-Mercapto-1,3,4-thiadiazol-2-yl)-2-phenylacetamide derivatives (3a-3j)*

According to the scheme 1, in a flat bottom flask appropriate phenylacetic acid derivative (1a-1j) was mixed with equimolar quantities of *N*-ethyl-*N*-dimethylaminopropyl carbodiimide (EDC) and hydroxybenzotriazole (HOBt) in acetonitrile solvent, and was stirred for 30 minutes at room temperature. Then, Equimolar quantity of 5-amino-1,3,4-thiadiazole-2-thiol (2) was added to the reaction medium. The stirring was continued for 24 h. After confirmation of the reaction end by TLC, the acetonitrile was evaporated and 20 mL of water and 20 mL of ethyl acetate were added to the residue. The organic phase was separated and was washed two times by sodium bicarbonate 5%, diluted sulfuric acid and brine. Anhydrous sodium sulfate was added, filtered and the ethyl acetate was evaporated. The obtained powder was washed by diethyl ether, *n*-hexane and purified by column chromatography (EtOAc/Petroleum ether).

*2-(2-Fluorophenyl)-N-(5-mercapto-1,3,4-thiadiazol-2-yl)acetamide (3a)*

mp. 224 ^0^C, Yield: 63%, IR (KBr, cm^-1^) ῡ: 3151, 2931, 2804, 1712, 1570, 1492, 1465, 1342, 1311, 1234, 1180, 1145, 1068, 759, 694. MS (*m/z*, %): 296 (M^+^, 55), 133 (48), 109 (100), 83 (15). 

*2-(3-Fluorophenyl)-N-(5-mercapto-1,3,4-thiadiazol-2-yl)acetamide (3b)*

mp. 162 ^0^C, Yield: 78%, IR (KBr, cm^-1^) ῡ: 3147, 2924, 1708, 1570, 1485, 1458, 1307, 1253, 1138, 1068, 964, 767. MS (*m/z*, %): 296 (M^+^, 30), 133 (55), 109 (100), 83 (10). 

*2-(4-Fluorophenyl)-N-(5-mercapto-1,3,4-thiadiazol-2-yl)acetamide (3c)*

mp. 158-160 ^0^C, Yield: 72%, ^1^H NMR (DMSO-d_6_, 400 MHz) δ: 3.1 (brs, 1H, -SH), 3.71 (s, 2H, -CH_2_CO-), 7.01 (t, 4-fluorophenyl), 7.30 (t, 4-fluorophenyl), 12.3 (brs, NH). IR (KBr, cm^-1^) ῡ: 3140, 3100, 2924, 1963, 1554, 1504, 1296, 1220, 1120, 1062. MS (*m/z*, %): 269 (M^+^, 55), 133 (55), 109 (100), 83 (30), 59 (10). 

*2-(2-Chlorophenyl)-N-(5-mercapto-1,3,4-thiadiazol-2-yl)acetamide (3d):*

mp. 212-216 ^0^C, Yield: 82%, ^1^H NMR (DMSO-d_6_, 400 MHz) δ: 3.86 (s, 2H, -CH_2_CO-), 3.98 (s, SH), 7.26-7.58 (m, 2-Chlorophenyl), 7.85-7.91 (m, 2-Chlorophenyl), 10.15 (brs, NH). IR (KBr, cm^-1^) ῡ: 3147, 2924, 2862, 1708, 1570, 1469, 1307, 1145, 1064, 744. 

*2-(3-Chlorophenyl)-N-(5-mercapto-1,3,4-thiadiazol-2-yl)acetamide* *(3e)*

mp. 210 ^0^C, Yield: 46%, ^1^H NMR (DMSO-d_6_, 400 MHz) δ: 3.68 (s, 2H, -CH_2_CO-), 3.81 (s, SH), 7.48-7.52 (m, 1H, H_5_-3-Chlorophenyl), 7.49 (s, H_2_-3-Chlorophenyl), 7.81 (d, 1H, *J* = 8 Hz, H_6_-3-Chlorophenyl), 7.87 (d, 1H, *J* = 8 Hz, H_4_-3-Chlorophenyl), 11.22 (brs, NH). IR (KBr, cm^-1^) ῡ: 3151, 2927, 2804, 1701, 1573, 1469, 1311, 1222, 1153, 1064, 783, 682. 

*2-(4-Chlorophenyl)-N-(5-mercapto-1,3,4-thiadiazol-2-yl)acetamide* *(3f)*

mp. 174 ^0^C, Yield: 65%, ^1^H NMR (DMSO-d_6_, 200 MHz) δ: 3.73 (s, -SH), 4.86 (s, -CH_2_CO-), 7.14-8.29 (m, aromatic), 13.21 (s, NH). IR (KBr, cm^-1^) ῡ: 3444, 3137, 3095, 2850, 2718, 1687, 1551, 1519, 1492, 1352, 1089, 960, 818, 712. MS (*m/z*, %): 285 (M^+^, 3), 280 (45), 167 (94), 149 (100), 125 (12), 113 (15), 71 (20), 57 (20). 

*N-(5-Mercapto-1,3,4-thiadiazol-2-yl)-2-(2-methoxyphenyl)acetamide* *(3g)*

mp. 200 ^0^C, Yield: 47%, ^1^H NMR (DMSO-d_6_, 200 MHz) δ: 3.82 (s, 2H, -CH_2_CO-), 3.89 (s, SH), 3.95 (s, 3H, -OCH_3_), 7.02 (m, 2H, 2-Methoxyphenyl), 7.27-7.4 (m, 2H, 2-Methoxyphenyl), 11.44 (brs, NH).IR (KBr, cm^-1^) ῡ: 3468, 3147, 2931, 2835, 1701, 1570, 1492, 1462, 1303, 1246, 1145, 1064, 1026, 798, 752. MS (*m/z*, %): 281 (M^+^, 73), 148 (75), 133 (15), 121 (100), 91 (90), 65 (15).

*N-(5-Mercapto-1,3,4-thiadiazol-2-yl)-2-(3-methoxyphenyl)acetamide* *(3h)*

mp. 114 ^0^C, Yield: 65%, ^1^H NMR (DMSO-d_6_, 400 MHz) δ: 3.67 (s, SH), 3.79 (s, 2H, CH_2_CO-), 3.84 (s, 3H, -OCH_3_), 6.88 (m, 3-methoxyphenyl), 7.26-7.33 (m, 3-methoxyphenyl), 7.29 (s, H_2_-3- methoxyphenyl), 11.24 (s, NH). IR (KBr, cm^-1^) ῡ: 3410, 3147, 2927, 1701, 1570, 1489, 1465, 1307, 1261, 1149, 1064, 779, 759, 690.

*N-(5-Mercapto-1,3,4-thiadiazol-2-yl)-2-(4-methoxyphenyl)acetamide* *(3i)*

mp. 152 ^0^C, Yield: 79%, ^1^H NMR (DMSO-d_6_, 200 MHz) δ: 3.35 (s, 2H, -CH_2_CO-), 3.38 (s, -SH), 3.79 (s, 3H, -OCH_3_), 7.76 (d, *J* = 8 Hz, Phenyl), 8.02 (d, *J* = 8 Hz, Phenyl), 13.1 (brs, NH). MS (*m/z*, %): 281 (M^+^, 45), 238 (25), 148 (90), 121 (100), 91 (25), 78 (30). 

*2-(4-Bromophenyl)-N-(5-mercapto-1,3,4-thiadiazol-2-yl)acetamide* *(3j)*

mp. 198 ^0^C, Yield: 68%, ^1^H NMR (CDCl_3_, 400 MHz) δ: 3.67 (s, SH), 3.75 (s, 2H, -CH_2_CO-), 7.18 (d, H_2,6_-4-Bromophenyl), 7.47 (d, H_2,6_-4-Bromophenyl), 10.6 (brs, NH). IR (KBr, cm^-1^) ῡ: 3150, 2980, 2825, 1699, 1571, 1558, 1487, 1400, 1319, 1296, 1064, 1012, 788. MS (*m/z*, %): 331 (M^+^+2, 15), 329 (M^+^, 15), 198 (30), 196 (30), 171 (60), 169 (60), 133 (90), 89 (100), 63 (30).

*Cytotoxicity assay*

Synthesized derivatives of 1,3,4-thiadiazole (compounds 3a-3j) were tested for cytotoxic activity at 0.1-500 mcg/mL concentration in three human cancer cell lines of PC3 cell (prostate cancer), HT-29 (Colon cancer) and SKNMC (Neuroblastoma). Cells from different cell lines were seeded in 96-well plates at the density of 8000–10,000 viable cells per well and incubated for 48 hours to allow cell attachment. The cells were then incubated for another 48-96 hours (depends to cell cycle of each cell line) with various concentrations of compounds 3a-3j. Cells were then washed in PBS, and 20 μL of MTT (3-(4, 5-dimethylthiazol-2-yl)-2,5-diphenyl tetrazolium bromide solution (5 mg/mL) were added to each well. An additional 4 hours of incubation at 37 ^o^C were done, and then the medium was discarded. Dimethyl sulfoxide (60 μL) was added to each well, and the solution was vigorously mixed to dissolve the purple tetrazolium crystals. The absorbance of each well was measured by plate reader (Anthous 2020; Austria) at a test wavelength of 550 nm against a standard reference solution at 690 nm. The amount of produced purple formazan is proportional to the number of viable cells (26).
